# Audiovisual Interactions in Front and Rear Space

**DOI:** 10.3389/fpsyg.2018.00713

**Published:** 2018-05-15

**Authors:** Christopher Montagne, Yi Zhou

**Affiliations:** Laboratory of Auditory Computation & Neurophysiology, Department of Speech and Hearing Science, College of Health Solutions, Arizona State University, Tempe, AZ, United States

**Keywords:** spatial hearing, sound localization, audiovisual integration, multi-sensory, cross-modal bias, rear space

## Abstract

The human visual and auditory systems do not encode an entirely overlapped space when static head and body position are maintained. While visual capture of sound source location in the frontal field is known to be immediate and direct, visual influence in the rear auditory space behind the subject remains under-studied. In this study we investigated the influence of presenting frontal LED flashes on the perceived location of a phantom sound source generated using time-delay-based stereophony. Our results show that frontal visual stimuli affected auditory localization in two different ways – (1) auditory responses were laterally shifted (left or right) toward the location of the light stimulus and (2) auditory responses were more often in the frontal field. The observed visual effects do not adhere to the spatial rule of multisensory interaction with regard to the physical proximity of cues. Instead, the influence of visual cues interacted closely with front–back confusions in auditory localization. In particular, visually induced shift along the left–right direction occurred most often when an auditory stimulus was localized in the same (frontal) field as the light stimulus, even when the actual sound sources were presented from behind a subject. Increasing stimulus duration (from 15-ms to 50-ms) significantly mitigated the rates of front–back confusion and the associated effects of visual stimuli. These findings suggest that concurrent visual stimulation elicits a strong frontal bias in auditory localization and confirm that temporal integration plays an important role in decreasing front–back errors under conditions requiring multisensory spatial processing.

## Introduction

In everyday interactions with the environment, our initial reaction to the sudden onset of an unexpected sound (e.g., a quickly-passing vehicle) is to estimate its source location to better calibrate a reaction. The speed and accuracy of this reaction depend on the coordination of auditory and visual spatial functions. Auditory space is broad and extends to both front and rear space. In contrast, human vision is restricted to the frontal region and visual acuity declines quickly at peripheral locations away from the fovea (Curcio et al., 1990). It has been suggested that these differences in visual and auditory perceptual geometries necessitate a two-step process in sound source localization with an initial coarse, but broad, auditory detection, followed by a refined visual analysis (Perrott et al., 1990).

Visual stimuli can also influence auditory spatial judgment in immediate and direct ways. A well-known example is the “ventriloquist effect,” where visual cues capture the location of a sound to form a fused multisensory event (Jackson, 1953; Pick et al., 1969; Thurlow and Jack, 1973; Choe et al., 1975; Slutsky and Recanzone, 2001). Incomplete capture, or visual bias, could extend to auditory sources that are perceived as separated events (Welch and Warren, 1980; Bertelson and Radeau, 1981; Hairston et al., 2003; Wallace et al., 2004; Körding et al., 2007). Historically, due to a vision-centered approach in multisensory research, our knowledge about cross-modal spatial bias is limited to audio–visual (AV) interactions in the frontal hemifield. Whether or not the spatial information of visual stimuli affects the perceived origin of a sound source outside the field of vision is relatively less studied. Early observations of Jack and Thurlow (1973) showed that visual inputs presented directly from front (TV monitor) could capture the location of a speech sound presented straight from behind. However, to our knowledge, no systematic inquiry has been reported about the characteristics of the front–back interaction between vision and audition. Whether frontal visual cues can interact with the perceived back auditory events remains an interesting question not just scientifically, but as an ecologically important consideration for everyday life.

**FIGURE 1:**
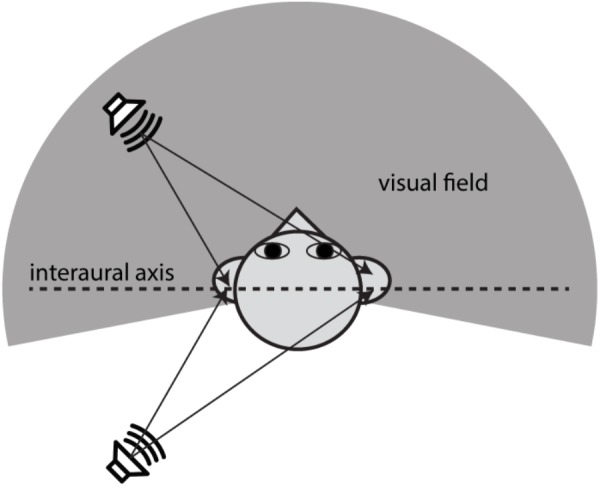
Illustration of the acoustic origin of the front–back confusion and the geometry difference between auditory and visual space. When the positions of two sound sources are mirror-symmetric along the interaural axis, the sound incidences from them will reach the ear canals with the same ITDs. This symmetry in binaural cue distribution is the cause of front–back confusions in sound localization. The auditory space embraces both frontal and rear fields, whereas the visual field is limited largely to the frontal field. This study addresses the question whether vision affects the front–back confusion in sound localization.

Evaluating front–back discrimination and related errors in auditory localization tasks requires careful consideration of auditory spatial mechanisms. This study focused on two types of auditory localization errors that have been widely addressed in the literature: (1) lateral or local errors refer to responses that are deviated away, in no systematic manner, from the actual sound source location and (2) front–back errors refer to responses at approximately the correct angular displacement relative to the midline but in the wrong front–back hemifield (Carlile et al., 1997; Macpherson and Middlebrooks, 2000). Interaural time and level differences (ITDs and ILDs) offer the primary information about the horizontal angle of a sound source (e.g., see Middlebrooks and Green, 1991). Front–back errors are rooted in the fact that these binaural difference cues do not, on their own, correspond with only one sound source location. For example, **Figure 1** shows that sounds coming from a fixed angle off the midline produce the same ITDs, regardless of whether they originate from the front or rear space. Actually, there is a host of angular directions which provide the same ITD information; they constitute what is known as the “cones of confusion,” (see a review by Blauert, 1997). Because of the symmetry of cue distribution, front–back confusions (FBCs) are a common error in sound localization. When listeners are not moving, resolving FBCs typically requires the usage of spectral cues from the pinna filtering that contributes to elevation localization. Since elevation-sensitive spectral cues are mostly above 5 kHz in humans (Middlebrooks and Green, 1991), the FBC rate depends on the spectral content of a stimulus and low-mid frequency sounds (<4000 Hz) evoke more front–back confusions than do high-frequency sounds (Stevens and Newman, 1936; Burger, 1958; Makous and Middlebrooks, 1990).

Front–back confusionss are not localization errors that arise from “noisy” auditory processing (non-systematic error). Instead, FBCs are a systematic error caused by the fact that a given set of binaural cues specify not one, but a range of sound source locations (i.e., cones of confusion). As such, FBCs cannot be explained by a simple analysis of the distribution of binaural difference cues. It is therefore important to distinguish FBCs from other errors in localization response, e.g., lateral or local errors. Macpherson and Middlebrooks (2000) offered an appropriate analysis method that took this difference between FBCs and other localization errors into account. Using an iterative regression procedure, they categorized the auditory localization responses on the horizontal plane into three categories: (1) quasi-veridical responses retain not only the front–back, but also the left–right direction of a sound source; (2) reversed (front–back confused) responses retain largely the lateral angle (within ±45°) of a sound source but reflected to the opposite hemisphere; (3) spurious responses are the remaining responses with large errors (more than 45°) in the lateral angle of a sound source independent of front–back accuracy. To our knowledge, few previous studies have examined whether visual bias affects differently the FBC and spurious response rates in auditory localization.

The current study investigated audiovisual interactions in front and rear space. We used time-delay-based stereophony to generate a fused, “phantom” sound source located between two concealed loudspeakers. Auditory fusion due to “summing localization,” the basis of stereophony, is a salient perceptual phenomenon (Blauert, 1997) underlying successful applications of sound reproduction and entertainment systems (e.g., TV and theater). The stereophony paradigm provides a simple platform to test how multisensory cues interact in a *perceptual* space, as previously suggested by others (Van Wanrooij et al., 2009), using the phantom sound sources. This stimulus paradigm also allows us to test how cue reliability in the auditory domain affects the strength of multisensory integration. Our previous work showed that time-delay-based stereophony in the frontal field is subject to strong visual bias due to the broadening of an auditory image with conflicting ITD and ILD information (Montagne and Zhou, 2016). The current study extended our previous work and included the rear auditory space in the testing apparatus. Based on the analysis method developed by Macpherson and Middlebrooks (2000), we characterized the stereo localization responses into quasi-veridical, front–back confused, and spurious responses. We found that when broadband noises and LED flashes were simultaneously presented to a subject, visual stimuli enhanced the frontal bias of perceived sound source locations and increased FBC errors for rear auditory targets. Presenting visual stimuli also increased the spurious error rate by shifting the lateral direction of a sound source toward the LED direction. Interestingly, this lateral shift predominantly applied to perceived frontal sounds. The associated visual effects on front–back confusion rate, but not spurious error rate, increased with decreased stimulus duration, suggesting that temporal integration affects localization errors in domain-specific ways.

## Materials and Methods

### Participants

Fifteen students from Arizona State University (5 female, 10 male, ages 18–26 years, mean 22 years) participated in this study. All participants had a self-reported normal hearing and a normal or corrected-to-normal vision. Participants provided written informed consent and received financial compensation for their participation. All procedures were approved by Arizona State University’s Institutional Review Board.

### Apparatus

**FIGURE 2:**
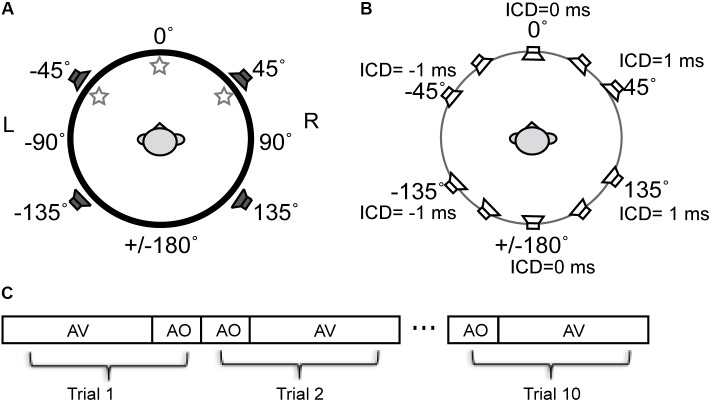
Schematic diagram of the experimental setup for stereophonic and visual stimuli used in experiments. **(A)** The spatial arrangement of auditory and visual cue sources described in the ±180° circular range. The negative and positive angles correspond to the left and right fields, respectively. Three LED lights were positioned at –45° (left-front), 0° (center-front), and 45° (right-front) at the subject’s eye level. Four loudspeakers were positioned at ±45° in front and ±135 in rear fields. **(B)** Time-delay-based stereophony configuration. The inter-channel delay (ICD) difference between left and right speakers was altered to shift the perceived azimuth of the phantom sound source. The perceived direction moves toward the leading speaker. For example, negative ICD moves the perceived sound location to the left side of the subject and positive moves the perceived sound location to the right side of the subject in both front and rear space. **(C)** Blocks of AO and AV stimuli were presented to a subject in random order. A total of 10 repeat was presented for each block.

The free-field sound localization task took place in a double-walled, sound-deadened chamber (Acoustic Systems RE-243, [2.1 m × 2.1 m × 1.9 m]) lined with 3^′′^ acoustic foam. The subject was seated in the center of the sound chamber. His/her head was stabilized using a high-precision head positioner (HeadLock^TM^, Arrington Research) equipped with bilateral head-fastener and chin-rest components. **Figure 2A** illustrates the spatial arrangement of loudspeakers and LED lights. In this circular arrangement, -90° corresponds to a position directly to the left of a subject, 0° to a position directly in front of the subject at midline, +90° to the right of the subject, and ±180° at the midline behind the subject. The current setup was identical to that used in our previous study (Montagne and Zhou, 2016) except for the two additional loudspeakers put in the rear sound field. The four loudspeakers were all hidden behind an acoustically transparent curtain and placed at lateral angles of ±45° in the frontal field and ±135° in the rear field at a distance of 1.1 m from the subject’s head. Three high-power LED lights (10 mm × 10 mm, six candelas) were positioned at -45° (left-front), 0° (center-front), and 45° (right-front) attached to the acoustic curtain at the subject’s eye level. White ping-pong balls encapsulated the LEDs to diffuse the light flashes. A touchscreen monitor was placed in front of the subject to record stimulus responses.

A custom MATLAB program generated auditory and visual stimuli and recorded subject responses. All digitized stimuli were sent through an external sound card (RME Multiface II) at a sampling rate of 96 kHz. The analog output ports were used for both auditory and visual stimuli. DC signals were directed through a simple transistor circuit to activate the LEDs. The precision of audio–visual stimulus synchrony was maintained at a sub-microsecond scale and was confirmed with oscilloscope measurements.

Auditory stimuli were frozen broadband white noise bursts with a pass-band from 50 to 50 kHz as determined by the frequency response range of the loudspeakers (Adam F5, full-range studio monitor). The noise waveform was gated in the Matlab program by a rectangular window of either a 15-ms or 50-ms duration. Different noise tokens were used at the two different stimulus durations. The rms amplitude of individual channel of the stereo signals was adjusted to match the power of the single-speaker control signals. The average intensity for all auditory stimuli (single-speaker and stereo signals) was maintained at 65 dB SPL (dBA) as verified using a sound level meter (Brüel and Kjær 2250-L) positioned at the location of subject head. Visual stimuli were 15 ms or 50 ms light flashes generated from one LED at a time. In audio–visual trials, auditory and visual stimuli were turned on and off at the same time and lasted for the same duration.

Sound stimuli were presented using time-delay-based stereophony to create a single apparent sound source. When the two loudspeakers emit identical sounds with a sub-millisecond time delay, the subject perceives a phantom sound source in between the pair of loudspeakers (Leakey, 1959). In this study, we controlled the position of the phantom sound by altering the time delay between the left and right loudspeaker signals either in front or behind the subject (**Figure 2B**). The inter-channel delays (ICDs) were -1, -0.5, 0, 0.5, and 1 ms. Negative ICDs generated a single perceived sound source to the left side of the subject and positive ICDs generated a source to the right side of the subject. Because substantial localization errors could occur when a stereo pair of speakers are in the same lateral field (Theile and Plenge, 1977), no phantom sound sources were generated between the front left and back left speakers, as well as between front right and back right speakers.

### Procedure

Stimuli were presented in randomized blocks of audio-only (AO) and audio–visual (AV) stimuli (**Figure 2C**). The AO block contained 14 stimuli. They were 5 phantom sound source positions between the front pair of speakers, 5 phantom sound source positions between the back pair of speakers, and 4 single speaker control signals (two front and two back). For the AV block, each of the auditory stimuli was paired with three LEDs in random order, resulting in a total of 42 AV stimuli (14 A stimuli × 3 V stimuli). The AO and AV blocks were each presented ten times in random order, resulting in a total of 560 trials.

Subjects indicated their perceived sound source location via a graphical user interface (GUI) on a touch screen monitor (10^′′^ × 8^′′^). The psychophysical method used was single-interval forced choice. Upon beginning the task, subjects triggered a trial by tapping the “Next” button on the GUI. After a stimulus ended, the participant indicated the perceived direction of the sound source by tapping one of 16 buttons, which were numerically labeled and positioned with a spacing of 22.5° along a circle on the GUI. Note that the stimulus and response choices are not mapped one-to-one, and subjects could choose a response location outside the lateral angles of four loudspeakers in front and back. This arrangement allowed us to construct the circular vector of responses (from -180 to +180°) to avoid edge effects, where responses would start to accumulate at the extreme ends of a range of response choices.

Before the test, subjects were asked to practice the testing procedure using a training panel for as long as they wanted. The purpose of the training was to allow a subject to pair up a response button with a perceived sound direction. Unlike the testing panel, where the subject had to choose one of the response buttons after the stimulus was presented, during training the subject could choose a response button to trigger a stimulus from the desired location. The training panel displays 10 buttons arranged on a circle. This includes 5 buttons that mark the locations from -45 to +45° in front and 5 buttons that mark the locations from -135 to 135° in back with 22.5° spacing. Since we did not know the exact perceived lateral positions of the generated phantom sound source stimuli for each subject, single-speaker stimuli were presented from the actual loudspeakers for the boundary locations at -45, +45, -135, and +135° provided by our setup. Within this boundary, the front (0°) and back (±180°) midline directions triggered the stereo stimuli with zero ICDs and the remaining intermediate choice locations triggered stereo stimuli with ±0.5 ms ICDs in front and back. The training procedure did not leave a subject with an impression that there were only ten potential source locations. As shown in results, responses to ±1-ms ICD, which were not included in training, frequently deviated from the theoretical positions of ±45 and ±135°. This deviation necessitates the usage of a full circular range for registering subject’s response in experiments.

The experiment lasted for 40–60 min, depending on the participant. At the beginning of a task, subjects were given specific verbal instructions to maintain a center-fixation before a trial started and to indicate the location of the sound they heard, not the light they saw. They were asked to keep their eyes open during the trial. While no specific instruction was given as to whether to look toward the heard sound direction, we cannot verify the gaze movement of a subject before, during, and after a trial since eye movement was not monitored. Subjects were encouraged to take breaks every 15 min. Subjects were not provided any feedback or knowledge of their results during or after the experiments. They were unaware of the total number of loudspeakers and spatial location of each speaker.

### Data Analysis

The circular angle of a response was calculated by mapping response buttons “1” through “16” to angles from -90 to -112.5° in 22.5° increments. Frontal responses are labeled between -90 and 90° and back responses between -91 and +91° from left to right. The sound localization responses were then partitioned into the AO condition and three AV conditions with left LED (AV_L_), middle LED (AV_M_), and right LED (AV_R_), respectively. For each AO and AV condition, we plotted the stimulus-response confusion matrices between circular angels of responses and stimulus ICDs for each subject.

To differentiate the front–back and lateral errors in the data, responses were classified using an iterative regression procedure developed by Macpherson and Middlebrooks (2000). This procedure was used because the localization data had a two-peak distribution due to front–back reversals and contained a non-trivial number of outliers, which made the standard least-squares linear regression method inappropriate. The first goal of the iterative regression procedure was to obtain the boundaries for front and back response regions that would be counted as correct. Based on the results of this analysis, responses were classified as front–back confusions or spurious results (i.e., incorrect responses due to lateral error). For the first iteration of the procedure, the dataset was limited to response points that fell in the correct (front or rear) hemisphere and these points were linearly regressed. Next, points deleted in the first iteration were returned. Data points lying ±40° away from the regression line were deleted and the remaining data points were regressed again. This procedure was repeated until convergence, usually for two to three iterations, and the final linear estimate was used to classify response regions. We classified the localization responses into three categories: quasi-veridical, front–back confused, and spurious. Quasi-veridical responses were the data points within ±40° of the regression line. Front–back confused responses were the data points within ±40° of the regression line but reflected into the opposite (front or back) hemisphere of the stimulus. In this study, our calculation of FBC combined front-to-back and back-to-front error rates. The remaining data points were classified as spurious responses. These procedures closely follow those defined in Macpherson and Middlebrooks (2000) with the exception that we used ±40° criterion to isolate quasi-veridical and FBC responses, instead of the ±45° range used in their study. After sorting our response types, we calculated the percentages of front–back confused and spurious responses for each of the AO and AV conditions for each subject. A percentage reflects the ratio of the total number of error responses to the total number of stimuli presented at all ICDs.

To analyze the influence of visual cues in each response category, the percentages of quasi-veridical, front–back confused, and spurious responses from AO conditions was subtracted from their respective percentages of responses from the three AV conditions for each subject. The resulting difference in percentages was used to evaluate the degree of visual bias. The group mean and its standard error were also calculated for this statistic across all subjects and reported in Section “Results.”

To visualize and describe the changes in front–back confusion and left–right shifts of localization responses, we used the iterative Expectation-Maximization (EM) algorithm to fit the raw localization data (including all trials from all subjects) with a Gaussian Mixture Model (GMM) (McLachlan and Peel, 2004). This fitting optimization function is included in the MATLAB Statistics and Machine Learning Toolbox (The MathWorks, 2017). The GMM fits were estimated using a 1° resolution and better allowed the visualization of any AV influence on the entire data distribution.

We parameterized the fitting function by assuming the GMM consists of two components that account for the two clusters of responses, respectively. By choosing two components, the GMM model evaluates the mixing ratio of two Gaussian functions in fitting two response clusters with individual means and standard deviations (SDs). The GMM is preferable than the single-peak Gaussian model because the GMM can fit data with either one or two clusters by altering the mixing ratio. In other words, it is inclusive of situations with no or many FBC in responses. By contrast, the single-peak Gaussian model cannot adequately handle the FBC data, and it reports an averaged, inappropriate overall distribution between frontal and rear response clusters.

To confirm that the two-component (or two-peak) GMM was indeed more adequate than a one-component (or single-peak) Gaussian model, we used the Akaike Information Criterion (AIC) (Akaike, 1998) as a basis for selecting the optimal number of components for the model while avoiding over-fitting. Lower AIC values indicate better goodness-of-fit of a statistical model. Although more components could yield even better fit in some stimulus conditions, we chose not to extend the model with three or more peaks to avoid over-fitting based on the relatively low horizontal resolution of the responses. The modeling results show that the AIC values were consistently lower for two-peak GMM than the single-peak model for all auditory stimuli under all light conditions (10 ICD × 4 lighting × 2 duration = 80 conditions). In addition, we tested the significance of the goodness-of-fit for both models using the χ^2^ statistic. The GMM model provided an overall excellent fit for all auditory stimuli under all light conditions (*p* > 0.79, 79 out of 80 conditions). In contrast, the single-peak model failed to predict the data clusters with FBC (*p* < 0.001, 60 out of 80 conditions).

## Results

### Front–Back Confusions in Stereo Spatial Perception

All subjects participated in this study were able to identify the lateral angle of a stereo sound source in the AO condition and their response typically shifted from left to right as the ICD changed from -1 ms to 1 ms. With the exception of only one subject’s response to 50-ms duration stimuli, stereo localization results were significantly correlated with ICD for both front (*p* < 10^-5^, median *R*^2^ = 0.723 for 15-ms and median *R*^2^= 0.818 for 50-ms stimuli) and back (*p* < 10^-5^, median *R*^2^= 0.775 for 15-ms and median *R*^2^= 0.803 for 50-ms stimuli) stimuli; linear regression. Despite this consistency in lateral localization performance, the subjects as a whole showed various degrees of front–back confusion, generally with more front–back confusions for short-duration stimuli.

**FIGURE 3:**
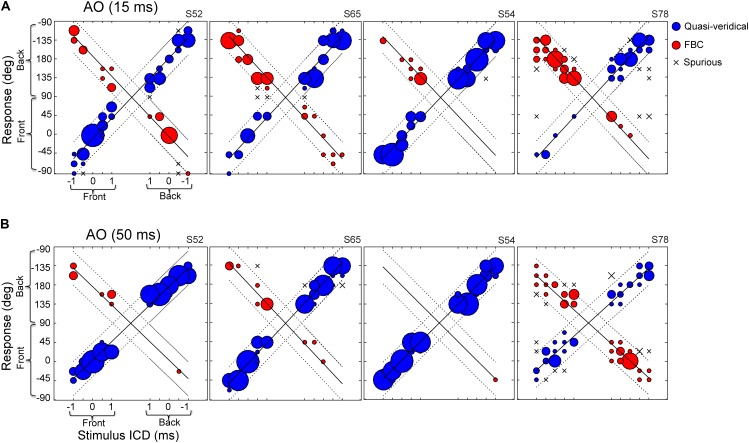
Stereo localization in front and rear space by four exemplar subjects in the AO condition. **(A)** Results for 15-ms noise burst. **(B)** Results for 50-ms noise burst. The left–right direction of a perceived sound source was altered by increasing/decreasing the inter-channel delay (ICD from –1 to 1 ms) between two loudspeaker signals. On the each panel, the left half shows front stimuli and the right half shows rear stimuli; the bottom half shows frontal response (–90 to 90°) and top half shows rear response (91 to –91°). See **Figure 2** for the graphic layout of the setup. The size of a symbol is proportional to the percent response for a given stimulus. Regression lines were fit to the data using an iterative regression procedure (see section “Materials and Methods”). Localization responses were grouped into three categories: quasi-veridical, front–back confused, and spurious. The dotted lines on each plot bound the quasi-veridical (positive slope) and front–back confused (negative slope) response regions within ±40° of regression results.

**Figure 3** shows the responses of four example subjects to the same broadband noise burst presented at durations of 15-ms (**Figure 3A**) and 50-ms (**Figure 3B**). Of the subjects we tested, the performances of S52, S54, and S65 were typical of most subjects, while S78 showed pronounced front–back confusions. For the 15-ms condition (**Figure 3A**), S52, S65, and S54 all localized accurately, showing high incidences of quasi-veridical responses (blue) and low incidence of spurious responses (“x”). Yet, their responses were often front–back confused (red). Similar to earlier findings by others (Makous and Middlebrooks, 1990; Wightman and Kistler, 1999), the two types of FBC confusions, front-to-back and back-to-front, did not always occur at the same rate. For example, S65 and S54 produced accurate responses for rear hemi-field targets, but they frequently confused the frontal targets as originating from the back. Of all subjects we tested, S78 displayed the greatest number of front–back confused responses with a strong bias toward the back.

The results from the 50-ms condition (**Figure 3B**) revealed the stimulus-duration effect on front–back confusions. With a longer duration stimulus, all subjects produced more quasi-veridical responses and fewer front–back confused and spurious responses. S54 demonstrated this trend most explicitly, as front–back confusions were almost eliminated. Increasing stimulus duration also mitigated the response bias toward the back of the poor localizer, S78. At the population level, increasing stimulus duration significantly reduced the FBC rates for most subjects (13 out of 15 for stereo and 10 out of 15 for single-speaker control).

**FIGURE 4:**
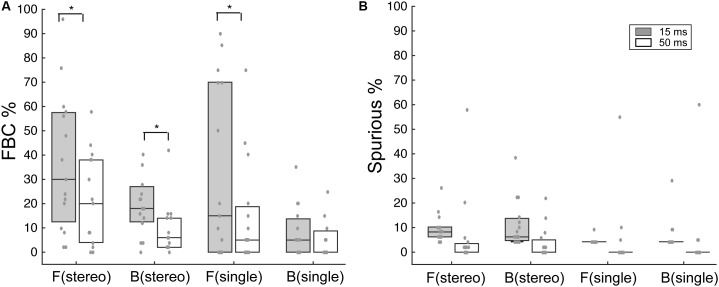
Analysis of FBC and spurious rates for stereo and control stimuli. **(A)** Boxplots of the upper, median, and lower quartiles of the FBC rates of all subject data for frontal and rear stimuli. Individual data (gray dots) are superimposed on each quartile distribution. Higher FBC for the 15-ms condition was found for frontal and rear stereo, as well as frontal control stimuli. **(B)** Same analyses on the spurious rate for frontal and rear stimuli. No significant difference was found between 15- and 50-ms conditions. SEM, standard error of the mean. ^∗^*p* < 0.05, Wilcoxon signed rank test.

**Figure 4** summarizes the overall FBC rate for all subjects (*N* = 15). In the data analysis, we separately analyzed FBC for front and back stimuli to minimize the effects of individual front/back bias on the group average data. As shown in **Figure 4A**, there is a wide range of the FBC rate among subjects for both stereo and single-speaker control stimuli. The population averaged FBC rate dropped significantly from 15-ms to 50-ms conditions for both front (*p* = 0.037) and back (*p* = 0.016) stereo stimuli; Wilcoxon signed rank test. On the other hand, only frontal control stimuli showed a significant decrease in FBCs with increased stimulus duration and rear control stimuli caused less FBC in our results (front, *p* = 0.034; back *p* = 0.33; Wilcoxon signed rank test). We also questioned whether the spurious responses, which characterize large lateral localization errors, would follow the same trend. As shown in **Figure 4B**, the spurious rates were small (medians are less than 8%) and did not significantly change with duration in any conditions (*p* > 0.05; Wilcoxon signed rank test).

### Visual Capture in Front–Back and Left–Right Judgment

**FIGURE 5:**
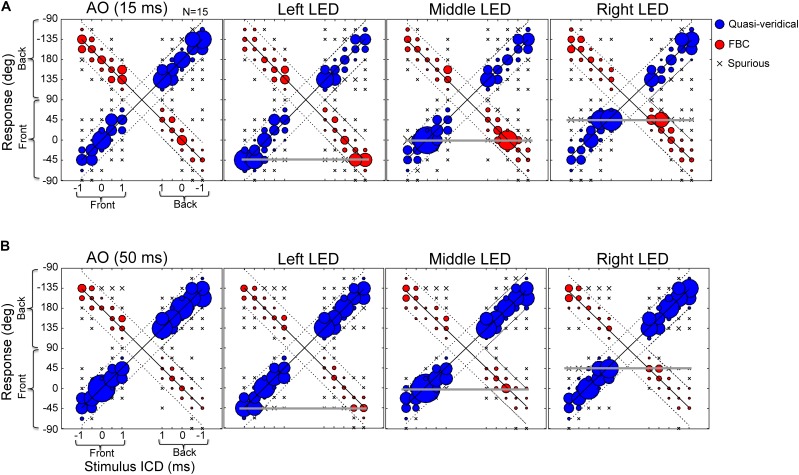
Effects of LED lights on stereo localization. **(A)** Results of 15-ms stimulus duration with and without lights. **(B)** Results of 50-ms stimulus duration with and without lights. The three response categories (quasi-veridical, front–back confused, and spurious) are similarly plotted as those in **Figure 3**. The stimulus-response layout on each panel is identical to those shown in **Figure 3**. Briefly, the left half of a panel shows front stimuli and the right half shows rear stimuli; the bottom half of a panel shows frontal response (–90 to 90**°**) and the top half shows rear response (91 to –91**°**). The three horizontal lines mark the locations of left, middle, and right LED lights (located at –45, 0, and 45**°**, respectively). Visual capture is manifested by an increased bobble size relative to AO results on each of the three horizontal lines.

Next, we investigated whether visual stimuli that are exclusively located in the frontal field can modulate sound localization in rear space. **Figure 5** shows the population average of the raw stereo localization results with and without visual stimuli at 15-ms (**Figure 5A**) and 50-ms (**Figure 5B**) conditions. The data are arranged and plotted in the same manner as those in **Figure 3** except that the three visual conditions (LED lights located at -45, 0, and 45°, respectively) are added. These response distributions were estimated from all trials of all subjects; no within-subject averaging was conducted, so instances of front-to-back and back-to-front confusions are preserved.

Compared to the auditory-alone condition (AO, left column), there is an increased instance of responses at the location of presented light stimuli (marked by horizontal gray lines). This effect applied to both frontal and rear auditory stimuli. More specifically, after visual stimulation, responses that were localized in the rear hemi-field, either quasi-veridical or front–back confused, could “flip” to the frontal field. Comparing the 15-ms and 50-ms condition, stimulus duration also affects visual bias. Frontal visual bias becomes weaker with increasing stimulus duration. Since sound and light stimuli were always turned on and off together, the decreased visual bias at the 50-ms condition is not due to prolonged auditory stimulation over visual stimulation.

These results show that interactions between frontal vision and rear sound fields can occur in auditory spatial judgment. Since early AV experiments often focused on the left–right interactions between auditory and visual targets restricted to the frontal visual field, we further examined whether the changes in FBC caused by frontal visual stimuli follows similar cross-modal principles, such as enhancement of interaction through increased spatial congruency (Stein and Meredith, 1993). In this analysis, we fitted the population data (as shown in **Figure 5**) with a two-peak Gaussian function at each ICD (See details in Methods on Gaussian Mixture Model, GMM). We used this function because it allowed us to analyze the distributions of both quasi-veridical and FBC responses as well as their lateral shifts over a closed, 360° circle. Since the GMM fits were estimated using a 1° resolution, it also allowed us to better capture the overall change in the shape of data distribution, which was sampled at a 22.5° resolution.

**FIGURE 6:**
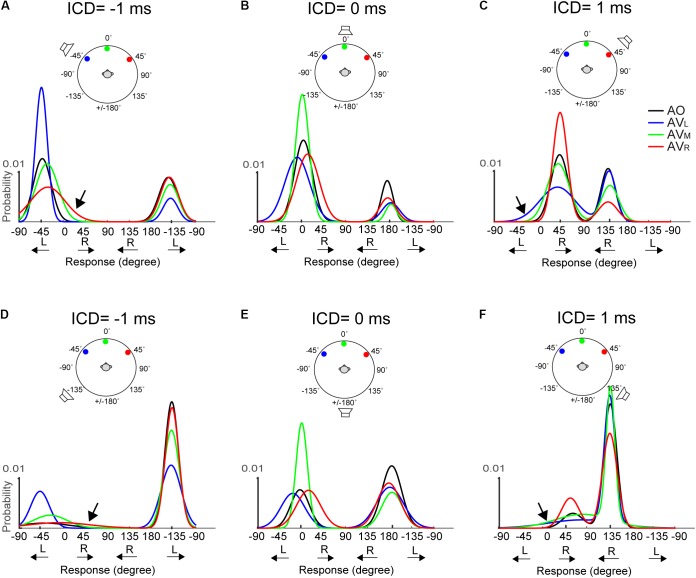
Two-peak Gaussian functions for AO and AV responses at the 15-ms condition. The top **(A–C)** and bottom **(D–F)** rows show the results obtained using the two frontal or rear speakers, respectively. Each curve was obtained by fitting the data from all trials and all subjects using the Gaussian Mixture Model (see section “Materials and Methods” for detail). The stimulus configuration in terms of ICD and lighting conditions is illustrated above the results on each panel; the speaker sign marks the quasi-vertical response position. The AO results (black) show that at ICD of –1 ms, the responses were clustered on the left side at –45 and –135 degrees, at ICD of 0 ms, the responses were clustered on the midline at 0 and ±180 degrees, and at ICD of 1 ms, the responses were clustered on the right side at 45 and 135 degrees. The color lines show changes in the left–right and front–back responses after adding visual stimulation.

**Figure 6** shows the fitted Gaussian functions for AO and AV results at -1, 0, and +1 ms ICDs for the 15-ms stimuli. Results at ±0.5 ms ICD, which are not shown, exhibit similar trends. The top and bottom rows show the results obtained using the two frontal or rear speakers, respectively. Plotting in this format, a front–back confused response is marked by the response peak on the opposite front–back hemifield than the actual speaker locations. This includes the conditions at which the FBC response was in the rear but the stimulus was from front (e.g., responses centered around -135° in **Figure 6A**) and the conditions at which the FBC response was in front but the stimulus was from back (e.g., responses centered around -45° in **Figure 6D**).

We note two inter-related trends in subjects’ responses. First, a back-to-front switch in response mostly occurs when A and V stimuli were presented from the same side (left or right). Take, for example, the condition shown in **Figure 6A**, in the AO condition (black), where frontal stereo inputs generated leftward stereo responses centered at -45° (front, quasi-vertical) and -135° (rear, front–back confused). In the AV conditions, left light (blue) caused a large change in front–back choices, resulting in much reduced leftward FBC responses at -135° in the rear space. By contrast, the same leftward FBC responses show almost no changes to right (red) light relative to the AO results. This means that visual stimuli do not simply “pull” rear auditory responses to the front. This is evidenced by the fact that there is little interaction between frontal visual and rear auditory stimuli if they are on the opposite side of the midline. Almost mirror-symmetric patterns are seen when the light was presented from the right (**Figure 6C**). More interestingly, the same lateral dependence of FBC is observed when the auditory signals were presented from the rear space (**Figures 6D–F**).

Second, the left–right shift in response occurs only when the auditory stimulus was *perceived* to originate from the frontal space and the magnitude of the shift is stronger when the light was originated from the opposite lateral field to the perceived sound direction. Examine the AV results shown in **Figure 6A** again. Right (red) light caused noticeably rightward shifts of the frontal response distribution at -45° (marked by arrow), whereas no such lateral shifts were observed in the back at -135°. Similar mirror-symmetric shifts are seen in **Figure 6C** with left light (blue). Our reasoning that this phenomenon applied only to perceived frontal responses is supported more strongly by the results to rear stimuli as shown in **Figures 6D–F**. Here, more rear responses (quasi-vertical) were observed than the frontal responses (front–back confused) in the AO condition. But the rear response distributions do not appear to skew toward either left or right light directions after visual stimulation. Because instances of frontal responses are rather low in **Figures 6D,F**, the lateral shift is hard to inspect from the fitted Gaussian curves. Still, a small but noticeable shift can be seen in those due to right light (red) at -45° in **Figure 6D** and left light (blue) at 45° in **Figure 6F**.

The midline auditory responses evoked by 0-ms ICD showed combined changes in both FBC and lateral position (**Figures 6B,E**). For all three visual stimulus locations, the responses to the rear hemifield at ±180° do not exhibit any systematic left–right shift with the light position. In contrast, there is a strong lateral shift toward the position of the light stimulus for frontal responses around 0°, regardless of whether these responses are quasi-veridical or front–back confused.

**FIGURE 7:**
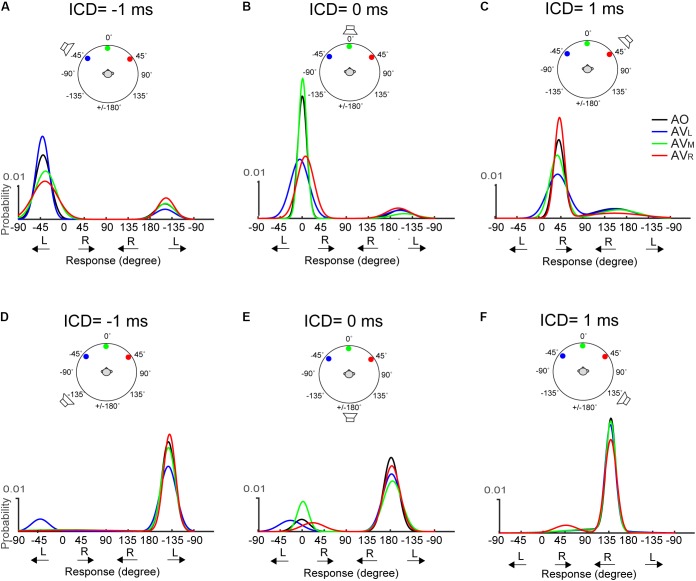
Two-peak Gaussian functions for the front and rear responses in AO and AV conditions at the 50-ms condition. The top **(A–C)** and bottom **(D–F)** rows show the results obtained using the two frontal or rear speakers, respectively. The data is presented in the identical format as those in **Figure 6**.

The above-mentioned, two visual effects do not appear to change with stimulus duration. **Figure 7** shows results for the 50-ms condition. The responses at 0-ms ICD, i.e., at midline, (**Figures 7B,E**) reveal very similar trends to those shown in **Figure 6**. However, lateral shifts of FBC responses are difficult to inspect due to their infrequency (e.g., **Figures 7D,F**).

To estimate the significance of the observation that the left–right shift occurs predominantly for *perceived*, frontal auditory events, we conducted a two-way analysis of variance (ANOVA) with the lighting condition (AO, AV_L_, AV_M_, AV_R_) as one factor and the sound location (through changing ICD between -1, -0.5, 0, +0.5, and +1 ms) as the second factor. ANOVA was conducted on the complete dataset shown in **Figure 5**. The responses were categorized first into four quadrants based on front/back directions of stimulus/response (front and back directions are marked on the ordinate and abscissa in **Figure 5**). The separation of different stimulus/response quadrants removes the effects of FBC and allows us to focus on visual modulation along the left–right direction only. **Table 1** shows the ANOVA performed on each quadrant. Regardless of the front or back directions of stimuli or responses, the effect of sound location (i.e., ICD) is always significant. This confirmed again that subjects could perform stereo localization in both frontal and rear space. ANOVA also confirmed that only when the perceived sound location was in front was there a significant effect of visual stimulus direction. This observation applies to both 15-ms and 50-ms duration.

**Table 1:** Two-way ANOVA for stereo localization responses.

Response	Stimulus	Duration (ms)	Light location	Sound location (ICD)
Front	Front	15	*F*(3,19) = 17.62,	*F*(4,19) = 131.07,
			*p* < 1e-4	*p* < 1e-5
		50	*F*(3,19) = 8.5,	*F*(4,19) = 183.32,
			*p* = 0.0027	*p* < 1e-5
Front	Back	15	*F*(3,19) = 19.54,	*F*(4,19) = 149.37,
			*p* < 1e-4	*p* < 1e-5
		50	*F*(3,19) = 8.84,	*F*(4,19) = 39.56,
			*p* = 0.0023	*p* < 1e-5
Rear	Front	15	*F*(3,19) = 2.29,	*F*(4,19) = 454.75,
			*p* = 0.132	*p* < 1e-5
		50	*F*(3,19) = 0.57,	*F*(4,19) = 154.59,
			*p* = 0.6424	*p* < 1e-5
Rear	Back	15	*F*(3,19) = 1.9,	*F*(4,19) = 921.47,
			*p* = 0.1834	*p* < 1e-5
		50	*F*(3,19) = 0.73,	*F*(4,19) = 2287.6,
			*p* = 0.5545	*p* < 1e-5

*Results are partitioned based on frontal and rear dimensions of stimulus and response for 15- and 50-ms conditions. The averaged results across 15 subjects were used; we could not conduct repeated measures because FBC yielded uneven sample sizes on frontal and rear responses for the ANOVA analysis. Prior to the ANOVA analysis, we examined the normality of the data at each ICD (4 lighting × 5 ICD = 20 conditions); 15 out of 20 conditions for the 15-ms condition and 13 out of 20 conditions for the 50-ms condition were judged to follow a normal distribution (p > 0.05, Lillie-Test). The lack of sufficient data points at some ICDs (e.g., FBC responses at the 50-ms condition) is a main factor limiting the significance of the normality test.*

**FIGURE 8:**
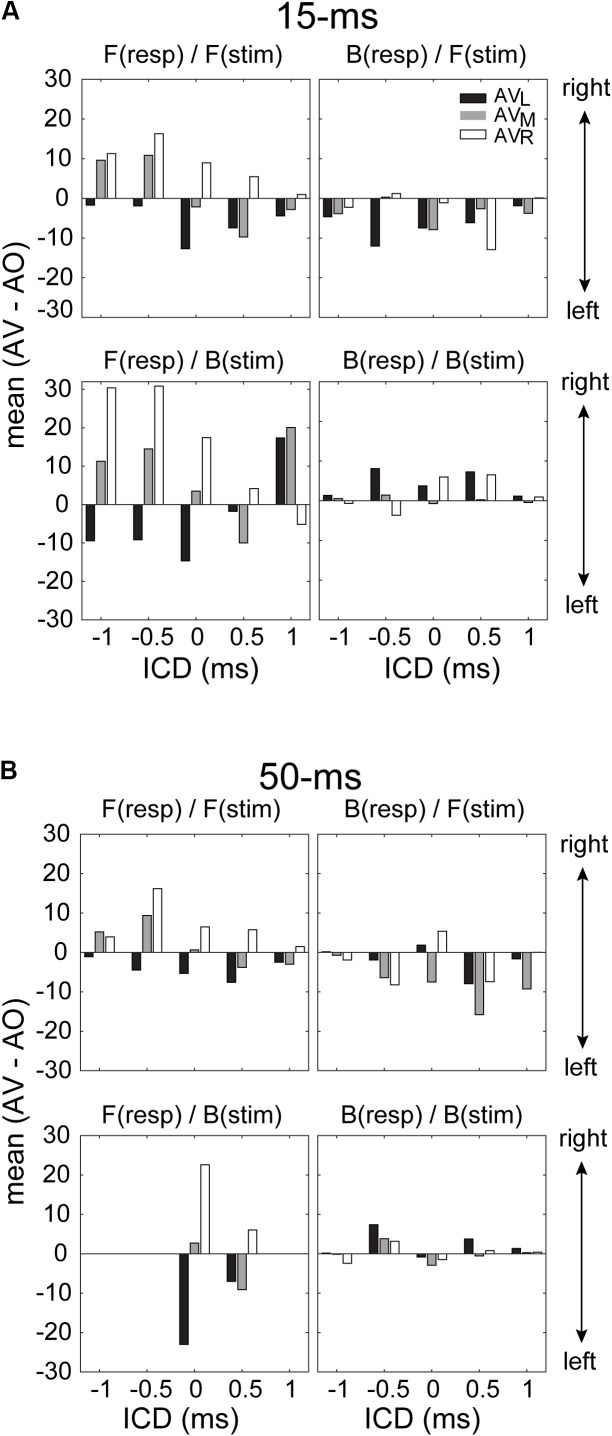
Magnitude of the lateral shift in stereo localization after visual stimulation for 15-ms **(A)** and 50-ms conditions **(B)**. The lateral shift was calculated as the difference between the means of the GMM functions for AV and AO responses shown in **Figures 6**, **7**. For the back responses, we remapped them to the angle range between –90° and 90° as those in front. For all conditions, negative and positive changes in the mean indicate leftward and rightward visual biases, respectively. The results for *F*(resp)/*F*(stim) at the 50-ms condition are in complete due to a lack of responses at some ICDs.

While the ANOVA confirmed the presence of visual influences on stereo localization in some, but not all, conditions, it does not reveal how the directional information of LED lights interacted with the directional information of the stereo sound source. To address this question, we quantified the patterns of visual modulation in the left–right direction on each of the four stimulus/response quadrants mentioned above. For this analysis, we extracted the difference in the mean of a GMM function between AO and AV results at each ICD. Since the responses were fitted with a two-peak GMM function, it was straightforward to obtain the means of frontal and rear responses. **Figure 8** shows the results for left, middle and right LEDs. The analysis confirmed that only frontal responses to either frontal or back stimuli showed the typical patterns of visual capture at both 15-ms and 50-ms conditions (two left panels in **Figures 8A,B**). That is, the perceived direction of a sound moved toward the direction of the light stimulus. The left and right LEDs caused either a leftward or rightward shift, respectively; the middle light caused either a leftward or rightward shift, depending on the relative positions of the sound and light. This applied to responses at most ICDs (with only one exception at 1-ms ICD from the bottom-left panel in **Figure 8A**). By contrast, the systematic variations of visual bias are largely absent for rear responses, independent of the light directions and stimulus duration (two right panels in **Figures 8A,B**).

**FIGURE 9:**
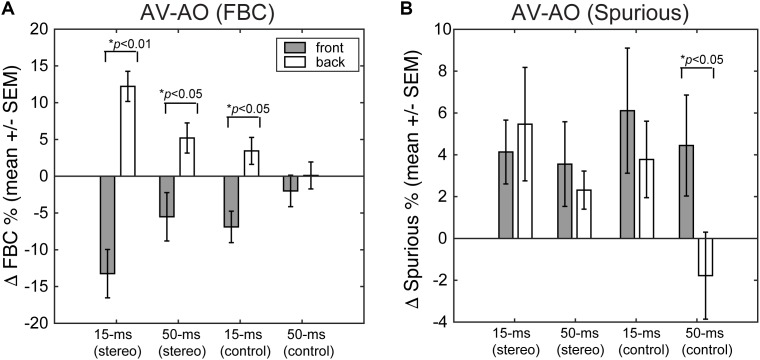
**(A)** Changes in FBC rate after visual stimulation. **(B)** Changes in spurious response rate after visual stimulation. For each error category, the data are contrasted between front and rear stimuli for both stereo and single-speaker control conditions.

Finally, we quantified the extent to which presenting a light stimulus from the frontal hemifield would change the two types of localization errors – FBCs and spurious localizations. **Figure 9** shows the population mean of changes in FBC (ΔFBC = FBC_AV_ – FBC_AO_, **Figure 9A**) and in spurious rate (ΔSpurious = SP_AV_ – SP_AO_, **Figure 9B**) averaged across ICD and lighting conditions. The data were partitioned based on the front/back source locations, stimulus duration, and stereo/control configurations.

The results show that visual stimuli tend to “pull” auditory responses toward the front. As such, the addition of visual cues led to decreased FBC rates for sounds presented from the front and increased FBC rates for sounds presented from behind (**Figure 9A**). This change in FBC was significant for both 15-ms and 50-ms stereo stimuli and 15-ms frontal control stimuli (front stereo, *p* = 0.002, back stereo *p* = 0.024, front control *p* = 0.04; paired *t*-test). For 50-ms control stimuli, the very small changes rendered non-significant differences between front and back (*p* = 0.368). Comparing the duration effect on visual influences, a larger change in FBC (either decreased or increased) was observed for 15-ms than 50-ms stimuli. But the difference was only significant for stereo stimuli (front stereo, *p* = 0.003, back stereo *p* = 0.008, front control *p* = 0.12; back control *p* = 0.138; paired *t*-test).

Examining the spurious rate (**Figure 9B**), we found that visual cues *increased* the error rates for all but the 50-ms control stimulus from back. No significant differences were found between front and back directions for stereo stimuli at either 15- or 50-ms duration (*p* > 0.25; paired *t*-test). This result is expected as visual cues caused large lateral shifts in localization responses (**Figures 6**, **7**) and these lateral shifts contributed to the spurious rate. The change for 50-ms control stimuli was significant (*p* = 0.036) as a result of a decreased spurious rate for sound presented from back during light stimulation.

## Discussion

This study investigated the influences of visual stimuli on sound localization in both front and rear space. The azimuth of a phantom sound source was varied through time-delay-based stereophony between a pair of loudspeakers in front or behind a subject. In comparison to left–right discrimination, front–back discrimination was highly sensitive to stimulus duration (**Figure 4**), and the occurrence of visual cues (**Figure 9**) that were synchronously presented with sound stimuli. For the short-duration (15 ms) noise burst stimuli, the back-to-front flip caused by visual cues significantly increased front–back confusions. Our results along with early reports by Jack and Thurlow (1973) demonstrate that vision could affect the auditory spatial judgment of a *perceived* frontal target, even though the source was actually behind the subject, due to front–back ambiguities (i.e., “cones of confusion”) inherent to auditory localization.

### Relations to Other Studies of Front–Back Errors in Sound Source Localization

As sound waves reach the ear canals, they interact with head/body geometry in complex ways. This interaction creates spectral cues, and binaural differences of time and intensity cues humans can use to estimate sound source location. For humans, the ITDs and ILDs are primarily used for sound localization in azimuth (Wightman and Kistler, 1992), whereas monaural spectral cues associated with pinnae filtering are used for sound localization in elevation, including front–back discrimination (Batteau, 1967; Blauert, 1969/1970; Musicant and Butler, 1984). Since the pinna’s surface geometry is the main factor causing the spectral modification of incoming sounds, front–back errors are highly variable among human subjects (Burger, 1958; Oldfield and Parker, 1984; Wightman and Kistler, 1989a,b; Makous and Middlebrooks, 1990; Carlile et al., 1997) and different subjects show different front or back preferences (Wightman and Kistler, 1999), presumably owing to usages of spectral signatures of one’s own ears (Middlebrooks, 1999).

The accuracy of elevation localization (including front–back discrimination) is also affected by acoustic factors bearing no directional information of a sound source on their own, such as stimulus duration (Hofman and Van Opstal, 1998; Macpherson and Middlebrooks, 2000; Vliegen and Van Opstal, 2004), signal-to-noise ratio (Good and Gilkey, 1996), and overall stimulus intensity (Hartmann and Rakerd, 1993; Vliegen and Van Opstal, 2004). In contrast, horizontal localization accuracy measured in the frontal field is largely immune to changes in stimulus level and duration (Yost, 2016). Increasing the stimulus duration of broadband noise burst from 3- to 100-ms can cause the FBC rate to drop from 30 to 50% to nearly zero for some subjects (e.g., see Figure 3 in Macpherson and Middlebrooks, 2000). The duration effect has been explained as evidence that spectral estimation requires temporal integration, which enables “multiple looks” of the long-term spectrum cues for elevation localization (Hofman and Van Opstal, 1998; Gai et al., 2013).

In this study, the perceived laterality of a stereo sound varies systematically with the inter-channel delay in both front and rear space (**Figure 3**). Front–back stereo localization revealed similar high individual variability and duration effects as those observed in early single-source studies. These similarities result from common localization cues used in stereo and single-speaker localization. As reported in our earlier study (Montagne and Zhou, 2016), the lateral extent of a stereo sound is governed by ITDs. Acoustic measurements confirmed (not shown) that this also applies to the rear sound field in our current setup, as a result of the symmetrical stereo setup. Similar to single-source stimulation, spectral patterns of a stereo sound also differ substantially between the front and rear fields (measurements not shown but explained below).

On the other hand, the inter-relationship between binaural and monaural spectral cues is different between single-source and stereo stimulation. Such disassociation assists us to gain new insights into the relationship between localization cue ambiguity and the engagement of visual dominance (discussed later). More specifically, in single-source stimulation, changing a source location will simultaneously alter binaural and monaural spectral cues. Functionally, the horizontal coding is not only governed by ITDs and ILDs, but also spectral cues. The role of spectral cues in horizontal localization is weak, but it can be strengthened after disruptions of binaural cues (Slattery and Middlebrooks, 1994; Van Wanrooij and Van Opstal, 2007; Kumpik et al., 2010; Irving and Moore, 2011; Agterberg et al., 2014). Under such situations (e.g., monaural hearing loss), the role of visual stimulation on left–right and front–back responses might be jointly affected by spectral information using single-source stimulation. This possibility needs to be further tested in future studies.

In contrast, because the stereo sound field was generated using only two speakers emitting slightly delayed, otherwise identical signals, the monaural spectra do not significantly differ between different perceived azimuths across ICDs and between left and right ears for stimuli delivered from speakers on the same front/back field. Rather, they are dominated by the signal spectrum from the speaker ipsilateral to that ear and the contribution of the signal spectrum from the contralateral speaker was significantly attenuated due to head shadowing. For example, when frontal stimuli were used, the monaural spectrum at the left ear, which remained largely unchanged with varying ICDs, followed the monaural spectrum to single, left speaker stimulation. This applied to monaural spectrum at the right ear as well except that it follows the monaural spectrum to single, right speaker stimulation. Because the two speakers in front were symmetrically positioned along the midline, the monaural spectra at left and right ears are largely consistent with each other. However, because the pinna filtering causes large spectral deviations between front and rear directions, the monaural spectra to frontal and rear stimulation are very different at the same ICDs. As a result, in the time-delay-based stereophony, the left–right and front–back discrimination are aided by separate and independent cues, namely, ITDs and monaural spectra. Such disassociation helps link visual bias with uncertainty or noise in the internal computation of auditory localizing cues before combining into a perceived auditory source location.

### Audiovisual Interaction in Front and Rear Space

The current study investigated how the acoustic features of auditory stimuli and its spatial dimension affect localization bias induced by visual stimuli. The results revealed that frontal visual stimuli influence auditory localization in two different ways: (1) shift the auditory responses in the left or right direction toward the light location when the auditory target was perceived to come from a frontal direction and (2) increase the rates of frontal over rear responses when the auditory and visual stimuli were perceived to come from the same side. As such, the directional information of visual stimuli appears to separately modulate the left–right and front–back estimation of a sound source, as opposed to its overall spatial location. In the latter case, we might expect to see visual capture of front–back responses independent of the lateral direction of a sound. However, as shown in **Figures 6**, **7**, a cross-midline, back to front response was seldom observed (e.g., a switch from a left+rear to right+front response with the right LED). This suggests that the observed interaction between auditory and visual information is not solely governed by the modality specific, source locations. Rather visual information may influence auditory spatial computation in domain-specific ways before the perceived sound location is established.

We found that front–back errors (**Figure 4**) and associated visual bias in FBC (**Figure 9**) were both higher for shorter duration stimuli. This observation is consistent with the cue reliability hypothesis (Battaglia et al., 2003; Alais and Burr, 2004; Ernst and Bülthoff, 2004) or “inverse effectiveness” (Stein and Meredith, 1993), which predicts a stronger cross-modal bias for the modality or stimulus conditions providing weaker evidence. In our experiments, a shorter stimulus duration may not allow adequate spectral estimation – the increased uncertainty in the front/back judgment makes it more conductive to visual bias. Our early study shows that the congruency between bilateral ITD and ILD cues affects localization certainty and visual basis in the left–right auditory localization (Montagne and Zhou, 2016). The currents finding suggests that spectral estimation (through temporal integration) and multimodal bias are also tightly connected in front–back, auditory localization. Together, they reveal that cue saliency within a modality is critical to the understanding of cross-modal bias.

While our results show clear interactions between frontal vision and rear audition, it is not straightforward to speculate on the principles of interactions between frontal vision and rear audition using existing Bayesian statistical models (Battaglia et al., 2003; Alais and Burr, 2004; Ernst and Bülthoff, 2004; Knill and Pouget, 2004), which are primarily based on the results of cross-modal perception of a seen target. Briefly, in these models, the position variable, *s*, has an implicit frontal origin [i.e., the prior distribution P(*s*)]. The modality-specific, sensory representation [i.e., likelihood estimates, P(A/*s*) and P(V/*s*)] consists of a one-to-one mapping of *s*, typically, in the form of a Gaussian function.

It is clear that when the front and rear spaces are both considered in the prior distribution, the assumption of a unimodal (or single-peaked) likelihood estimate immediately collapses. In our results (**Figures 6**, **7**), the quasi-veridical and front–back confused responses result in a bimodal likelihood function, P(A/*s*), which may be biased toward either front or rear space for different subjects (**Figure 3**). This bimodal likelihood function complicates the variance estimate and subsequently the construction of the posterior probability using combined auditory and visual estimates. Since previous AV Bayesian integration models do not account for FBC, the internal representation of sound location is often modeled using a single Gaussian (i.e., Alais and Burr, 2004). As shown in **Figures 6**, **7**, the GMM model adequately estimated the bimodal nature of auditory localization results with FBC and could serve as a promising, new approach for future generations of a probabilistic AV localization model.

As mentioned above, the left–right and front–back judgment of the stereo sound direction is governed by ITD and spectral cues, separately. In theory, the noise in spectral estimate could enhance front–back confusions and the uncertainty on binaural cue estimate due to conflicting ITD and ILD cues (Montagne and Zhou, 2016) could enhance left–right errors after visual stimulation. However, a direct application of the bimodal likelihood function is problematic in that it might violate the spatial rule in multisensory integration. This rule states that spatial congruency enhances the strength of audiovisual integration, and spatial incongruency encourages independent uni-sensory processing (Frens et al., 1995; Alais and Burr, 2004). Based on this spatial rule, frontal vision should not interact with rear auditory events, but it does.

Further scrutiny of our behavioral data suggests an alternative mode of interaction between vision and audition might occur when the stimulus space extends outside the field of vision. Our data show that the lateral shift of auditory localization only applied to a *perceived* frontal target (see also Van Wanrooij et al., 2009), whereas the enhanced frontal bias in FBC only occurred when the perceived sound source was on the same side as the LED flashes. Importantly, unlike the left–right shift, visual influences in front–back judgment do not result in a response at an intermediate location between A and V stimuli; rather it was a direct visual capture (**Figure 5**). This suggests that visual processing may interact with the left–right and front–back auditory judgment independently at the two different stages of a localization task: (1) an initial coarse and broad auditory detection to decide the relative front vs. back direction of an event and (2) refined visual analysis using integrated auditory and visual information if the perceived target location is in front.

According to the causal interference theory, the brain should limit the extent of integration between sensory events perceived to rise from different sources (Körding et al., 2007). The causality test likely occurs during the initial, auditory detection stage including front–back discrimination. If so, our data suggest that the perceptual geometry difference between vision and audition results in a frontal (visual) bias in the causality test. Our data also confirmed that after front–back judgment, audiovisual interaction occurs in the frontal space and vision calibrates the left–right direction of a perceived target based on known multisensory principles. For sound sources that were judged to be outside of the visual field, i.e., from rear space, visual cues are hardly effective.

### Effects of Visual Information and Eye Movement on Auditory Localization

Known as the “frame-of-reference” hypothesis, early studies argue that sound localization is more accurate when a listener can acquire, through free or voluntary eye movements, the knowledge of the spatial layout of a lighted environment (Thurlow and Kerr, 1970; Warren, 1970; Platt and Warren, 1972; Shelton and Searle, 1980). In their carefully designed experiments, Warren (1970) showed that active visual sensing of the physical layout of the environment and objects in it enhanced the acuity of listeners’ auditory localization. Importantly, Warren argued that eye movement *per se* does not improve the accuracy of auditory localization, but that an illuminated visual environment allows better visual-motor (eye-hand) coordination by providing a spatial reference to guide action. Recent studies have provided further support on the role of the visual-motor functions in auditory localizations. Results showed that directing gaze toward a sound can enhance auditory discrimination (Maddox et al., 2014) and fixed gaze direction during sound presentation (with a duration on the order of seconds) can shift the perceived sound direction to the opposite side of gaze for both frontal and rear targets (Lewald, 1998; Lewald and Ehrenstein, 2001).

In our experiments, the speakers were hidden and the spatial layout of the testing environment did not provide any landmark cues to aid sound source localization. Gaze direction is also unlikely a factor contributing to observed visual effects. We instructed subjects to maintain a central fixation at the beginning of each trial and register their responses on the response GUI after the stimuli were terminated. Subjects were not instructed to fixate or move their gaze toward a sound direction. Moreover, the auditory and LED stimuli we used were brief (15- and 50-ms), which are magnitudes shorter than those used to study the effects of free and fixed eye/gaze position (e.g., Lewald and Ehrenstein, 2001). Since the saccade latency of humans is mostly above 200 ms, depending on luminance and eccentricity of visual cues (Carpenter (1988), brief LED light we used is not a stable visual target that could guide eye movements to aid auditory localization. Nevertheless, since eye movement was not monitored, we cannot evaluate whether gaze positions evolve differently during stimulus presentation between perceived frontal vs. rear auditory events. This remains an interesting research question for future investigations.

## Author Contributions

CM and YZ designed the experiments and wrote the manuscript. CM conducted the experiments and performed the data analysis.

## Conflict of Interest Statement

The authors declare that the research was conducted in the absence of any commercial or financial relationships that could be construed as a potential conflict of interest.
